# Supplementary material for: Interpretable predictions from whole-body FDG-PET/CT using parameters associated with clinical outcome
Source: Commun Med (Lond). 2026 Apr 20;6:232. doi: 10.1038/s43856-026-01567-w (PMC13096647; doi:10.1038/s43856-026-01567-w)
Supplement: Supplementary file 2 — Supplementary Information [file 43856_2026_1567_MOESM2_ESM.pdf]

Supplementary Information

Comparison of DenseNet-121 vs 3D nnUNet

Supplementary Table 1: Comparison of 2D deep regression model and 3D segmentation model (nnUNet) across various criteria.

Comparison criteria	Deep regression vs Segmentation	
	2D DenseNet-121	3D nnUNet
[1] Total parameters	6,951,745	49,339,250
[2] Model size (MB)	84	250
[3] Training time per epoch (seconds)	68	260
[4] Validation time per epoch (seconds)	17	100
[5] Maximum batch size	30	1
[6] Input	2D projections	3D patches
[7] Overall performance	Good	Good
[8] RAM usage	low	high
[9] Operating speed	fast	slow

Saliency Analysis Success

TMTV

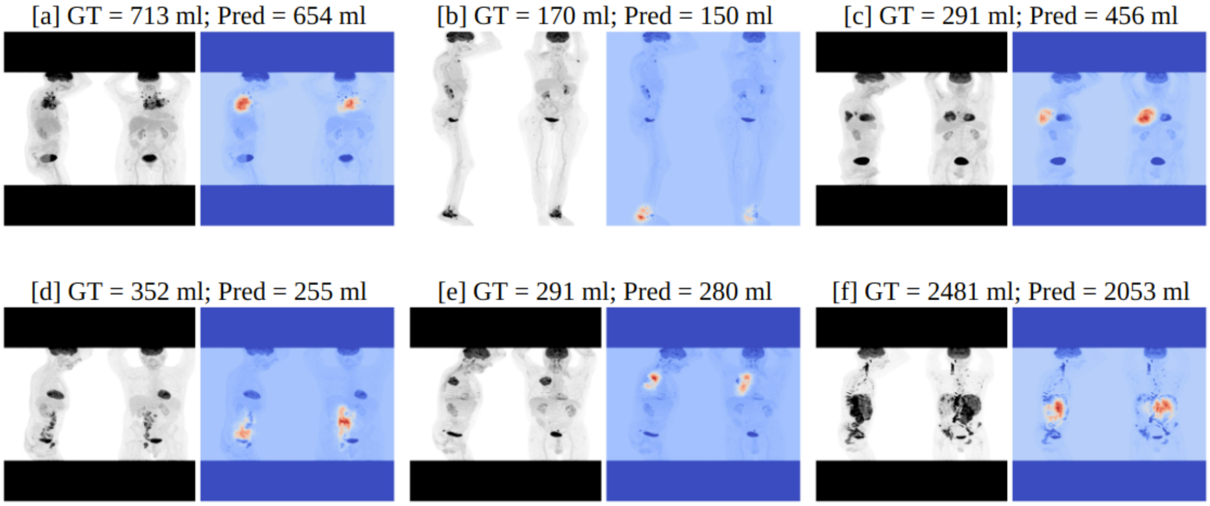

Supplementary Figure 1: Illustration of example cases from the best-performing model for Total metabolic tumor volume (TMTV) prediction (Pred) is shown in sub-figures [a] to [f]. The predicted TMTV values closely align with the ground truth (GT) TMTV. In each sub-figure, the left image represents the maximum intensity projection (MIP) of the Standardized Uptake Value (SUV) channel, while the right image displays the heatmap from the saliency analysis overlaid on the corresponding SUV_{orig}^{MIP}. The saliency maps primarily highlight the whole-body tumor regions that are the most influential for the TMTV prediction.

Age

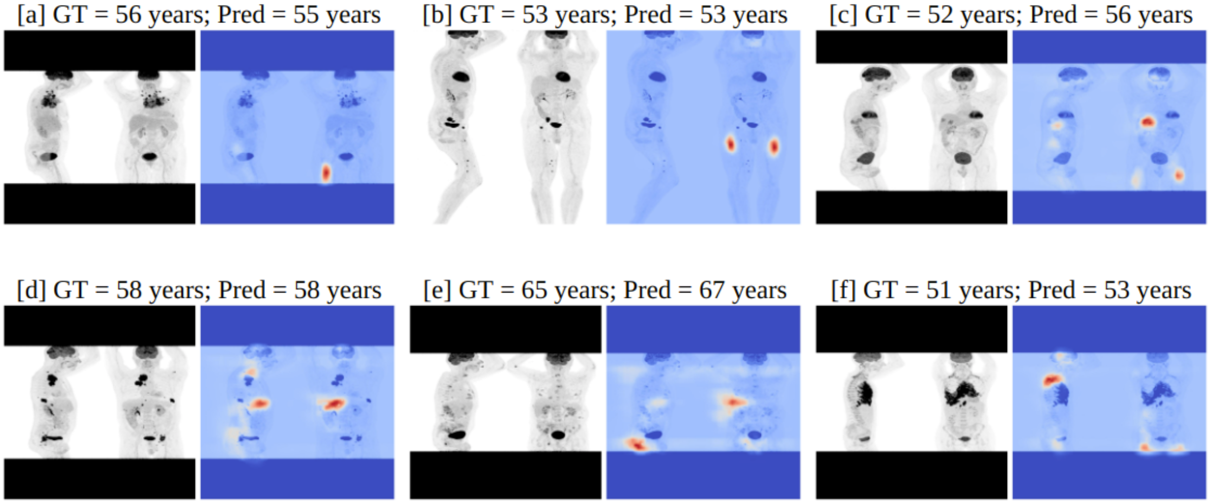

Supplementary Figure 2: Illustration of example cases from the best-performing model for age prediction (Pred) is shown in sub-figures [a] to [f]. In each sub-figure, the left image represents the maximum intensity projection (MIP) of the Standardized Uptake Value (SUV) channel, while the right image displays the heatmap from the saliency analysis overlaid on the corresponding SUV_{orig}^{MIP}. The predicted age closely aligns with the ground truth (GT) age. The saliency maps primarily highlight the following regions: the liver, thigh muscles, femur head, and pelvis, that are the most influential for the age prediction.

Sex

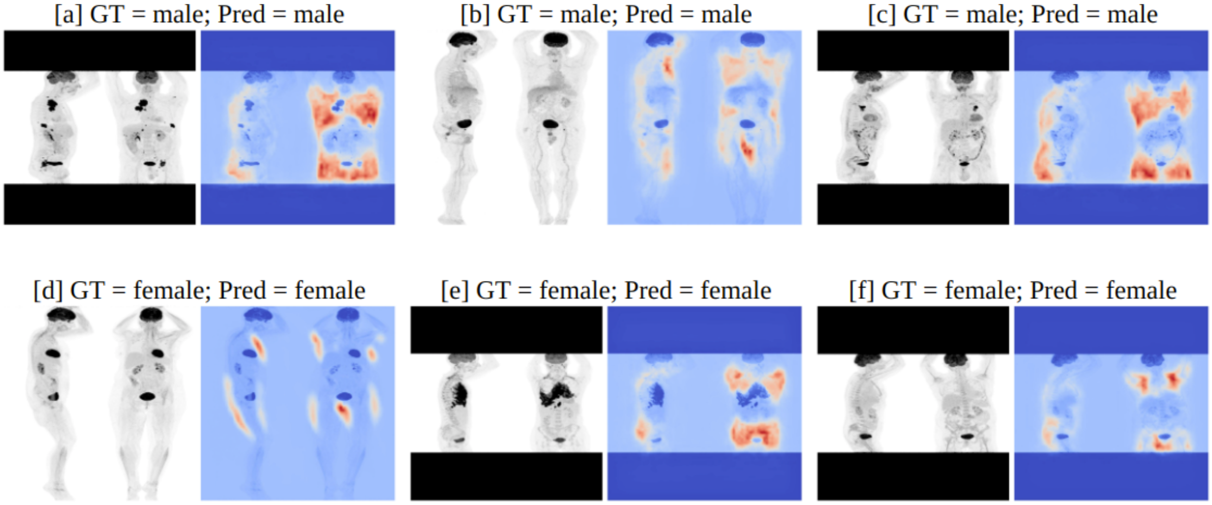

Supplementary Figure 3: Illustration of example cases from the best-performing model for sex classification is shown in sub-figures [a] to [f]. In each sub-figure, the left image represents the maximum intensity projection (MIP) of the Standardized Uptake Value (SUV) channel, while the right image displays the heatmap from the saliency analysis overlaid on the corresponding SUV_{orig}^{MIP}. The saliency maps primarily highlight the genitals and chest regions, that are the most influential for sex classification.

Diagnosis status

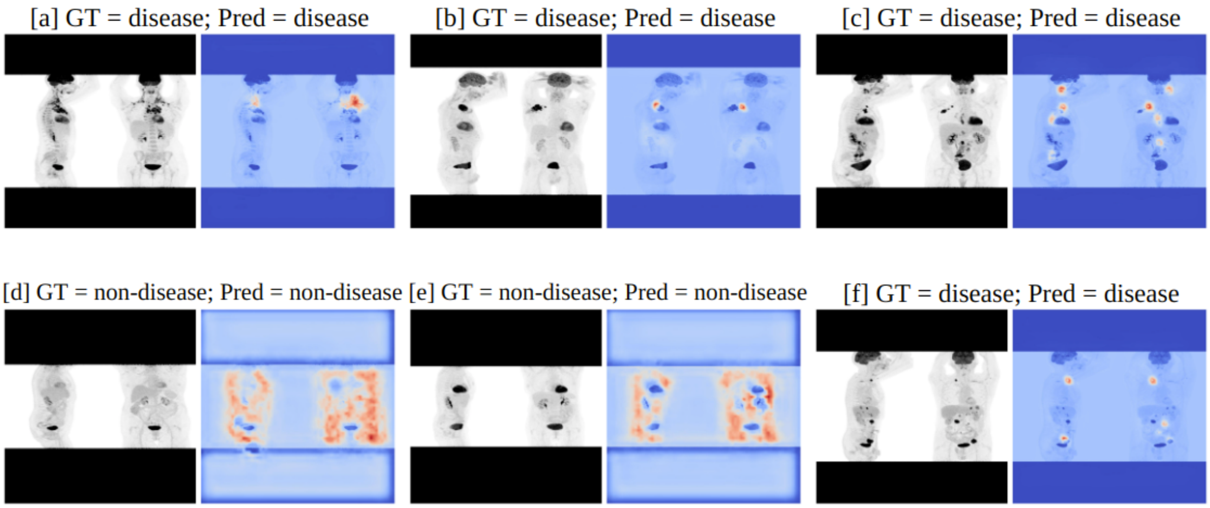

Supplementary Figure 4: Illustration of example cases from the best-performing model for diagnosis status classification is shown in sub-figures [a] to [f]. In each sub-figure, the left image represents the maximum intensity projection (MIP) of the Standardized Uptake Value (SUV) channel, while the right image displays the heatmap from the saliency analysis overlaid on the corresponding SUV_{orig}^{MIP}. The saliency maps primarily highlight the tumor regions in the whole-body, that are the most influential for diagnosis status classification.

Saliency Analysis Failures: Insights from Misleading Regions

TMTV

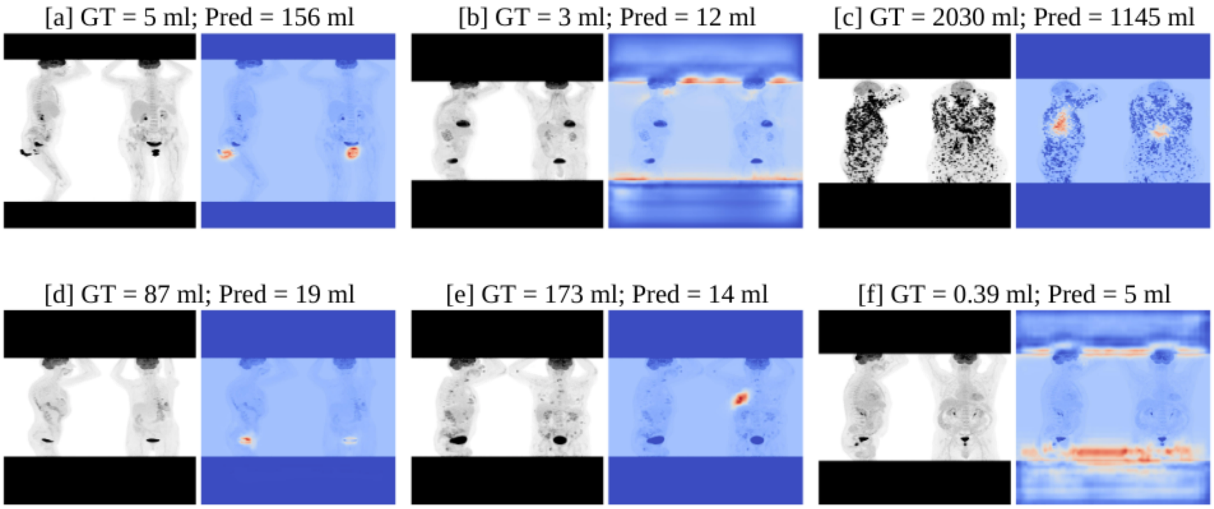

Supplementary Figure 5: Illustration of example cases where the model fails to accurately estimate TMTV is shown in sub-figures [a] to [f]. In each sub-figure, the left image represents the maximum intensity projection (MIP) of the Standardized Uptake Value (SUV) channel, while the right image displays the heatmap from the saliency analysis overlaid on the corresponding SUV_{orig}^{MIP}. When the model performs poorly, the saliency maps mostly highlight regions that either resemble tumors but are not (e.g. [a], [d]), or random areas when the actual tumor is too small and has poor contrast to be accurately detected (e.g. [b], [e], [f]).

Age

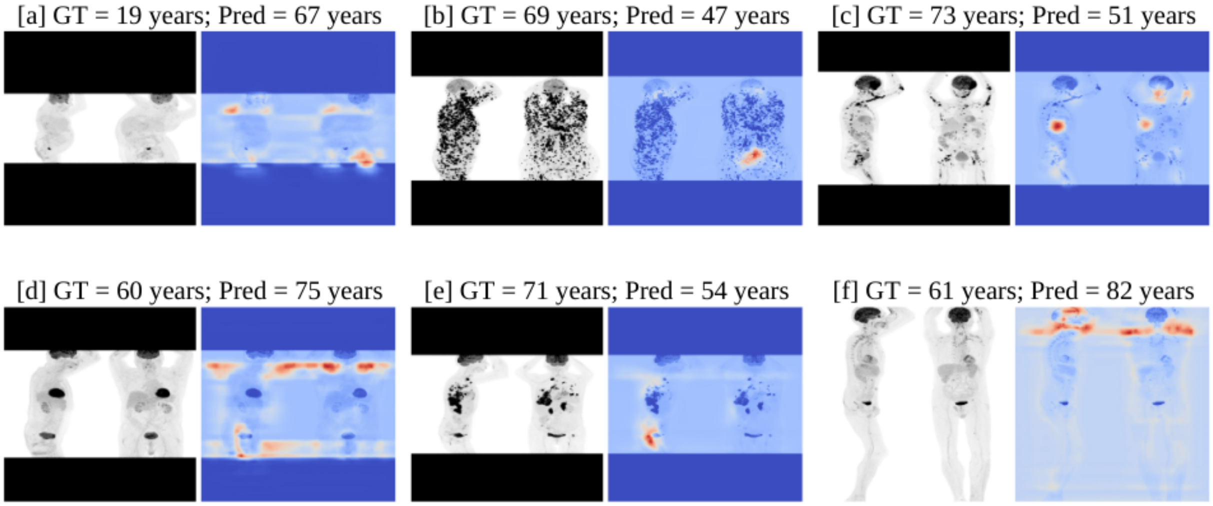

Supplementary Figure 6: Illustration of example cases where the model fails to accurately estimate age is shown in sub-figures [a] to [f]. In each sub-figure, the left image represents the maximum intensity projection (MIP) of the Standardized Uptake Value (SUV) channel, while the right image displays the heatmap from the saliency analysis overlaid on the corresponding SUV_{orig}^{MIP}. When the model performs poorly, the saliency maps appeared nonsensical. These cases typically involved issues with the input images, such as widespread tumors covering the entire body (e.g. [b], [c], [e]) or abnormal body structures (e.g. [a]).

Diagnosis status

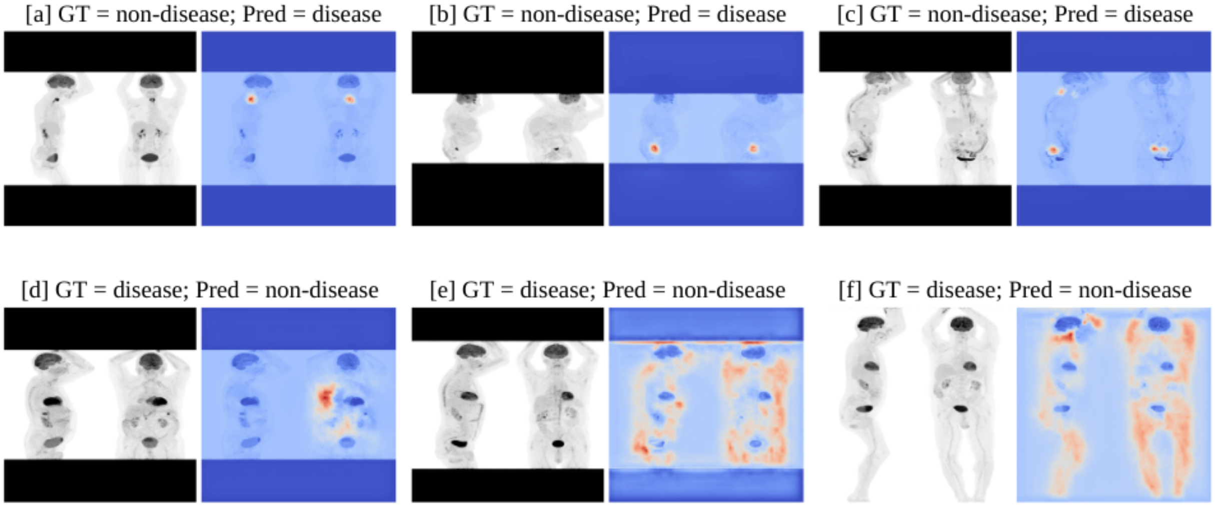

Supplementary Figure 7: Illustration of example cases where the model fails to accurately classify the diagnosis status is shown in sub-figures [a] to [f]. In each sub-figure, the left image represents the maximum intensity projection (MIP) of the Standardized Uptake Value (SUV) channel, while the right image displays the heatmap from the saliency analysis overlaid on the corresponding SUV_{orig}^{MIP}. When the model fails, the saliency maps mostly highlight regions that either resemble tumors but are not (e.g. [a], [b], [c]), or random areas when the actual tumor is too small and has poor contrast to be accurately detected (e.g. [d], [e], [f]).

606 **Ground truth tumor projections**

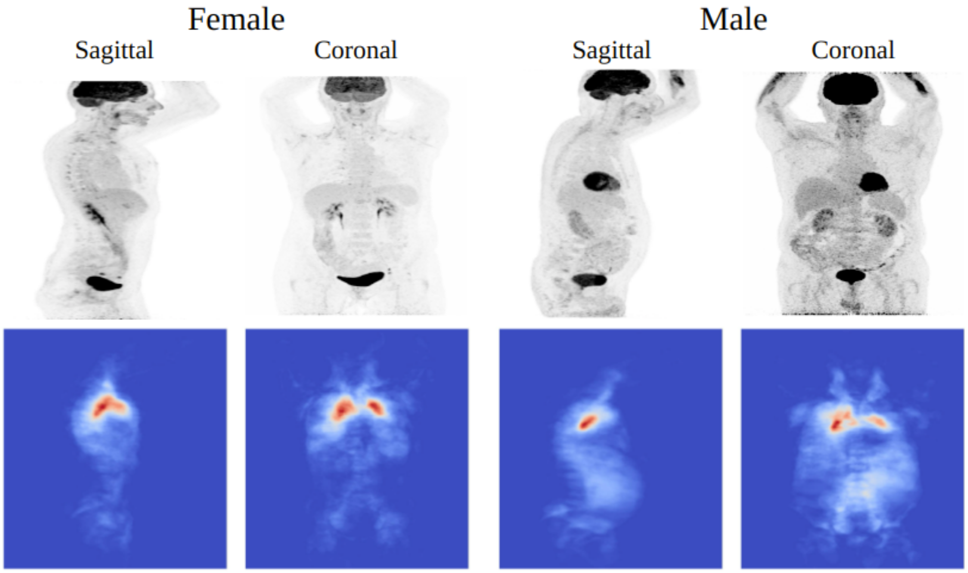

Supplementary Figure 8: Illustration of aggregated ground truth tumor segmentation projections along coronal and sagittal directions for males and females.
