# Supplementary material for: Interpretable predictions from whole-body FDG-PET/CT using parameters associated with clinical outcome
Source: Commun Med (Lond). 2026 Apr 20;6:232. doi: 10.1038/s43856-026-01567-w (PMC13096647; doi:10.1038/s43856-026-01567-w)
Supplement: Supplementary file 3 — Description of Additional Supplementary Files [file 43856_2026_1567_MOESM3_ESM.pdf]

Description of Additional Supplementary Files

File name: Supplementary Data 1

Description: Source Data for Figure 2.

File name: Supplementary Data 2

Description: Source Data for Figure 3.

File name: Supplementary Data 3

Description: Source Data for Figure 4 (a).

File name: Supplementary Data 4

Description: Source Data for Figure 4 (b).

File name: Supplementary Data 5

Description: Source Data for Figure 5 (a).

File name: Supplementary Data 6

Description: Source Data for Figure 5 (b).
